# Responsible autonomy: The interplay of autonomy, control and trust for knowledge professionals working remotely during COVID-19

**DOI:** 10.1177/0143831X221140156

**Published:** 2022-12-07

**Authors:** Neve Abgeller, Reinhard Bachmann, Tony Dobbins, Deirdre Anderson

**Affiliations:** Birmingham Business School, University of Birmingham, UK; School of Finance and Management, SOAS, University of London, UK; Birmingham Business School, University of Birmingham, UK; School of Management, Cranfield University, UK

**Keywords:** Control, COVID-19 pandemic, knowledge professionals, paradox, responsible autonomy, trust, working from home

## Abstract

This article revisits the concept of responsible autonomy, analysing the interplay of employee autonomy, management control and trust experienced by knowledge professionals in the UK compelled to work remotely during the coronavirus pandemic. The authors theorise about the tensions and paradoxes of responsible autonomy in the contemporary context of the COVID-19 crisis, drawing on empirical findings gathered in May 2020 and May 2021. Many participants experienced increased autonomy and discretion, but also work intensification and blurred work–life boundaries. Interestingly, many accepted this paradox as a palatable trade-off for the autonomy of being able to work from home, particularly where there was reciprocal trust between employee and manager. Trust is the glue in responsible autonomy, yet exists in tension with intrusive managerial control.

## Introduction

This article examines the concept of responsible autonomy (Friedman, 1977) by analysing the interplay of employee autonomy, management control and social relations of trust as experienced by knowledge professionals working remotely in the UK during the coronavirus pandemic. Governments around the world introduced lockdowns to combat the spread of the virus. The lockdown measures resulted in sudden and drastic changes in working conditions for millions of people in the UK and worldwide (Rofcanin and Anand, 2020; Unsworth, 2020), with many workers compelled to work from home (WFH) in March 2020 and followed by subsequent lockdowns during 2020–2021.

WFH is not a new concept, and a large body of knowledge already exists with regard to flexible working arrangements (FWAs) more broadly, and WFH in particular (e.g. Felstead and Henseke, 2017; Kelliher and Anderson, 2008, 2010). WFH and other FWAs were already practised prior to the COVID-19 pandemic in many knowledge-intensive industries. In fact, knowledge workers are seen as the ‘vanguards’ of a new era of work and employment relationships, and it is widely recognised that greater flexibility in their working arrangements has resulted in greater autonomy (Donnelly, 2009).

During the pandemic, however, WFH was no longer a choice, and millions were compelled by national governments to do so (Dobbins, 2021; Felstead, 2022). WFH on such a massive scale and under an extreme crisis had never occurred before (Unsworth, 2020). Drawing on in-depth interviews with 30 knowledge workers held in May 2020, with second-round focused interviews involving 21 of these participants in May 2021, in this article, we explore what this ‘new normal’ means to knowledge professionals specifically, elucidating their WFH experiences and interpreting them using the lens of responsible autonomy theory (Friedman, 1977). In doing so, the article theoretically views WFH during the context of lockdown as bringing about a sudden surge in responsible autonomy (Friedman, 1977), both as a form of work organisation experienced by workers and as a management control strategy requiring trust. Accordingly, our research question is: ‘How have knowledge professionals experienced the interplay of employee autonomy, management control and trust whilst working remotely during the COVID-19 pandemic?’ By analysing the dynamics of this interplay, we theoretically develop the concept of responsible autonomy to reveal some of the tensions and paradoxes relating to the experiences and consequences of knowledge professionals working remotely during this period.

In the rest of the article, we first discuss the challenges of WFH during the COVID-19 pandemic. We then conceptualise existing knowledge on responsible autonomy and control, including looking at the tensions and paradoxes associated with WFH, and consider trust as the glue in employment relationships. These thematic concepts constitute the theoretical foundation of our study. The next section outlines the abductive qualitative methodology, followed by presentation of findings relating to interlinking themes of responsible autonomy: employee autonomy, management control and trust, and associated tensions. Finally, contributions of the study are discussed.

## WFH during the COVID-19 pandemic

The circumstances of the COVID-19 pandemic and the absence of choice due to government-mandated WFH for most office workers are likely to have resulted in different experiences to those informing pre-pandemic research on flexible working. With COVID-19 spreading rapidly in 2020, employers did not have time to prepare themselves and their employees for such an extensive change in working arrangements. Being forced to undertake the largest WFH experiment ever, employers had to push aside possible concerns about working culture, lack of infrastructure or issues related to trust (Kramer and Kramer, 2020).

We can usefully draw from limited previous research studying WFH during natural disasters. For example, in an early study on this, Donnelly and Proctor-Thomson (2015) demonstrated that managing WFH during a crisis is shaped mainly by the turbulent external environment which is beyond the control of the organisation or its staff, availability of internalised work processes supporting WFH and an established certain level of employee autonomy, and preparedness of line managers to support their subordinates, both professionally and mentally.

Research during the early period of the pandemic illustrated that organisations with existing WFH experiences and associated values, such as employee-led flexibility and mutuality, coped better with the new conditions than those which did not have such experience (Bai et al., 2020). Moreover, transitioning to WFH during the pandemic went beyond the challenge of adapting to WFH requirements. For example, the pandemic has put significant psychological strain on individuals faced with dramatic changes in their behavioural routines. In some cases, the pandemic and associated lockdowns were reported to have serious negative effects on individuals linked to post-traumatic stress symptoms, confusion, frustration, anxiety, fear, uncertainty, isolation, etc. (Brooks et al., 2020; Unsworth, 2020). Furthermore, individuals with school-age children were also burdened with the daunting task of home schooling (Viner et al., 2020). Knowledge workers, many of whom can be considered to be relatively privileged, were most able to WFH. In contrast, blue-collar workers such as manual workers, those working in hourly paid contractual jobs and frontline essential or keyworkers performing critical roles during the pandemic were unable to WFH (Venkatesh, 2020).

Assuming both work and home responsibilities almost simultaneously, as required for knowledge workers at the onset of mandatory homeworking, is much more likely to lead to blurred work–family boundaries and therefore to role conflict, possibly eliciting stress and reducing work motivation (Rofcanin and Anand, 2020). Despite such negative consequences of lockdowns, recent research reveals that many employees welcomed the experience of WFH and developed a positive outlook linked to sentiments such as autonomy and trust (Dubey and Tripathi, 2020). Meanwhile, from an employer’s perspective, WFH may be perceived as reducing the scope for directly controlling and monitoring employees’ behaviour and productivity. Physical presence facilitates greater managerial control, both in terms of monitoring how tasks are fulfilled individually and how employees collaborate with co-workers (Felstead et al., 2003). Consequently, working virtually relies heavily on trust between employers/management and employees (Breuer et al., 2020).

Our research offers a unique perspective by shedding light on the interplay of responsible autonomy, encompassing the dynamics of employee autonomy, management control and trust relating to the experiences of knowledge professionals WFH during the pandemic. This is conceptualised next.

### Responsible autonomy and management control

As an influential scholar from the labour process tradition, Friedman (1977) distinguished between what he identified as two main alternative managerial strategies for coordinating the labour process, controlling workers and maintaining authority: direct control and responsible autonomy. Direct control refers to the close supervision, monitoring and surveillance of workers by management. By contrast, under responsible autonomy, management may allow or concede greater discretion to workers to judge and decide the organisation, timing and pace of work tasks, which entails a relaxation of direct management supervision. Contemporary research indicates that an increase in workers’ autonomy and discretion to organise their working lives is the strongest and most frequently identified benefit of flexible working patterns like remote working. Employees with the greatest autonomy over their work are the most satisfied with their jobs (Lopes et al., 2017; Masso, 2013; Wheatley, 2017).

Responsible autonomy, for skilled craft workers, can be traced back to the emergence of capitalism as a mode of production during the Industrial Revolution. Then, from the 1920s, early and subsequent human relations perspectives and ideas for ‘humanising work’ arose in the context of a backlash against inhumane Taylorist scientific management practices. Influenced by Tavistock Institute research in the 1950s and 1960s, this involved combining greater autonomy in the social relations of work with new advanced technologies and technical systems (Trist, 1963). Such ideas gained most traction in the Nordic ‘socio-technical’ and ‘quality of working life’ experiments from the 1960s (Enehaug, 2017; Hvid et al., 2010). Writing his labour process analysis in the 1970s from a Marxist perspective, Friedman (1977) saw responsible autonomy as increasingly emerging during that period due to an interplay between the labour process, worker resistance and evolution of managerial strategies of control. In essence, Friedman (1977) suggested that a responsible autonomy managerial control strategy stemmed from a need to accommodate worker resistance and shopfloor power in order to secure cooperation to maintain productivity in the labour process – notably when this coexisted with competitive pressures like labour scarcity and high product demand. However, the gradual shift towards responsible autonomy predicted by Friedman (1977) did not materialise more widely.

Also pertinent is that in clarifying the meaning of control, Friedman (1977) made an important distinction between conceptualising control in an absolute/overall sense to ‘identify those “in control”’, and in a more relative sense ‘to signify the degree of power people have to direct work’. Friedman (1977: 45) associated responsible autonomy with ‘the maintenance of managerial authority in an absolute or general sense by getting workers to identify with the competitive aims of the enterprise so that they will act “responsibly” with a minimum of supervision’. Edwards (1986) later made a similar distinction between detailed control and general control. The former refers to who controls specific decisions about how immediate work tasks are conducted; the latter covers the broader issue of securing workers’ consent to the overall organisational aims of their employers. Such analytical distinction between different levels of control in work organisations implies that managerial moves towards responsible autonomy and/or schemes for task-level employee participation leave employers in general control of decision-making because they retain overall power and authority over the work activities of subordinate employees. Subsequent empirical research reveals that the devolvement of responsible autonomy over the details of work organisation by management might enable them to achieve general control and authority in a wider sense (Geary and Dobbins, 2001). Additional discretion granted to employees may also encourage more self-disciplined workers if it fulfils some of their interests and work and performance expectations/outputs are clear (Edwards et al., 1998; Harley et al., 2010). Therefore, responsible autonomy may serve as a means of manufacturing consent at work (Burawoy, 1979).

This article provides a conceptual extension of the concept of responsible autonomy by analysing it as a pattern of work organisation that many employers were compelled to grant to knowledge professionals and other highly skilled employees; compulsory WFH patterns (where feasible) were imposed by governments in reaction to the external crisis of the pandemic. Unlike Friedman (1977), we do not see employers’ implementation of responsible autonomy as a deliberately intended managerial strategy, or stemming from a need to accommodate worker resistance. Rather, in the contagious COVID-19 virus emergency, responsible autonomy was a managerial control strategy that was a response to a situation suddenly and uniquely forced on employers. In a public health crisis, employers had little choice but to concede elements of detailed control over organisation of work to those employees that could WFH, and this was facilitated by new digital work technologies.

Relevant here is Friedman’s (1977) observation that those he called central skilled workers were more likely to experience responsible autonomy, and peripheral workers were more likely to be subjected to direct control. If we look at what has happened during the pandemic, knowledge professionals were more likely to have jobs that are more amenable to remote working/WFH, and therefore experience elements of responsible autonomy. By contrast, many essential frontline workers keeping society functioning during the pandemic could not WFH and were more likely to be subjected to direct control by management and exposed to possible health and safety risks. The COVID-19 pandemic magnified existing labour market polarisation and inequalities between those that had access to and choice over flexible working time arrangements (including remote working, WFH) and those that did not (OECD, 2021).

It is significant that Friedman (1977) discusses contradictions and tensions arising from responsible autonomy. To speak of contradiction does not mean responsible autonomy is not possible; it is to identify potential tensions, which are reconcilable or obscured to varying degrees. In attempting to extend responsible autonomy analytically in a contemporary setting, our article therefore considers possible contradictions, tensions and paradoxes arising from responsible autonomy in the context of the experiences of knowledge professionals WFH during the pandemic. For example, we explore the tensions in the relationship between autonomy from remote working and normative/peer control/online technological surveillance whereby colleagues demonstrate their online presenteeism to each other and management. In so doing, we add to understanding about the evolution of control in recent times.

### Flexible working: tensions and paradoxes

The outcomes of increased flexibility in working arrangements for both organisations and employees have been the subject of ongoing debate in academic research. Findings pertaining to FWAs from studies prior to the COVID-19 pandemic reveal positive outcomes like higher job satisfaction and organisational commitment (Kelliher and Anderson, 2008, 2010), lower turnover intention and reduced levels of role stress (Gajendran and Harrison, 2007). However, results are not overwhelmingly positive, as highlighted by Perrigino et al. (2018), who discuss the ‘dark side’ of work–life balance policies, particularly spillover or unintended consequences in the non-work domain, typically family.

It is noteworthy that previous research focused largely on situations where employees had some element of choice in where, when and how much they worked, in line with the widely accepted understanding of workplace flexibility (Hill et al., 2008). More recently, and in recognition of the contradictions evident in earlier research findings, there is greater focus on the tensions and paradoxical nature of workplace flexibility (Cañibano, 2019; De Vaujany et al., 2021; Putnam et al., 2014), especially in the context of knowledge work (Pérez-Zapata et al., 2016). Workplace flexibility is a paradox because it combines contradictory features or tensions. Firstly, the paradox relates to tensions in workers’ experiences and outcomes of flexible working. Second, in turn, this is linked to the more overarching paradox of a conflict of competing interests between employers’ instincts to maintain control over and monitor employee performance versus employees’ interests in greater autonomy and discretion to decide the organisation of their working day.

FWAs can be seen as an undertaking between employees and employers, and the management of constant tensions deriving from attempts to balance the employer-originated inducements and employee-provided contributions (Cañibano, 2019). Other tensions relating to the contested nature of FWAs include segmentation or integration of work and the rest of life (Falkenberg et al., 2020; Kossek et al., 2006), availability of FWAs versus access to them (Kossek, 2005) and involuntary versus voluntary flexibility (Kaduk et al., 2019).

For knowledge workers, tensions between autonomy and control have led to conceptualisation of the ‘autonomy paradox’, such that the more autonomy knowledge workers have, the more effort they put in, the longer the hours spent working, and the greater the blurring of boundaries between work and personal time (Mazmanian et al., 2013). Similarly, employees who experience autonomy specifically linked to WFH often work longer hours, or experience work intensification (Kelliher and Anderson, 2010). Enforced WFH during the pandemic has exacerbated pre-existing tensions and complexity. For example, Hassard and Morris (2022) offer the concept of work ‘extensification’ to denote two main changes to work: the stretching of work hours, often previously occurring within the workplace but now taking place within the home, and the breaching of the work–home boundary as many struggle with space within their homes and contemplate if they are WFH or indeed living at work. Moreover, De Vaujany et al. (2021) note that ethnographical research reveals many paradoxical experiences associated with remote working, with employee autonomy in tension with managerial technological surveillance and monitoring.

### Trust

The concept of trust almost completely escaped attention in debates on employment relationships and labour process theory when Friedman wrote his paper in the 1970s. A notable exception is Fox’s (1974) seminal typological distinction between low discretion/low trust and high discretion/high trust on a spectrum of managerial organisation of work and work orientations (see also Siebert et al., 2015). The former refers to direct control and the latter to responsible autonomy, with degrees of variance in between these ideal types. According to Fox (1974), increased division of labour under capitalism created routine low discretion work, with workers subject to more intense managerial control. The implication was mutual distrust and low trust dynamics in employment relations. However, there were contexts where Fox felt that high trust dynamics could emerge, notably among professionals in high discretion work roles characterised by high levels of autonomy, self-control and consent.

Nowadays, trust has been widely recognised as a key concept to analyse and understand nearly any social relationship, including employer–employee relations. Trust is commonly defined as willingness to accept vulnerability based on the expectations that the trustee will not intentionally harm the trustor and the risk of betrayal is low (Mayer et al., 1995; Rousseau et al., 1998). In other words, trust is inherently based on incomplete and fuzzy knowledge (Möllering, 2006). Trust can be used as a control strategy because it has moral implications. That is, a trustee will usually feel an obligation not to disappoint the trustor and to comply with their expectations. In much of the trust literature, however, trust is either seen as a complement or alternative social coordination mechanism to control (Costa and Bijlsma-Frankema, 2007; Long and Sitkin, 2018; Weibel, 2007). Whether an actor needs to decide between trust and control as a basis for a social relationship or whether trust and control go hand-in-hand in coordinating expectations and interaction between the two parties will depend on the specific circumstances and forms of trust and control. Especially where control and trust build on commonly accepted standards of behaviour rather than only on individual discretion, trust and control can occur in combination. By contrast, trust and control are difficult to reconcile in relationships where such standards are absent (Bachmann, 2001; Bachmann and Inkpen, 2011).

In the context of responsible autonomy, it seems clear that both trust and control play a significant role in the employer–employee relationship. WFH creates a situation where the employer needs to trust the employee but will not do so without any safeguards. Even if the employer is not controlling the work process in detail directly, the outcome of the work process will be subject to (overall) general control (Edwards, 1986; Friedman, 1977). The employee is in a similar position. He or she will usually trust the employer but only to a certain degree. Hence, WFH implies trust and control. Nonetheless, there can be serious conflicts arising from different interests between employer and employee regarding the interplay between trust and control. Trust is often simply portrayed as a positive unitarist (unity of interests) construct. Fox and other pluralist and radical scholars, however, acknowledge the tensions and conflicts of interest in the employment relationship which can erode trust and unleash a spiral of distrust (Fox, 1974; Siebert et al., 2015).

Under WFH conditions, the employer loses opportunities to directly control the employee and has to rely on trust more often than not at the level of more detailed work processes. At the same time, the employee may feel that they generally benefit through gaining more autonomy and discretion over their work. So, is the employee the winner who enjoys more trust and can avoid certain forms of unwelcome control? Is this finally the realisation of what Friedman meant by responsible autonomy and others who dreamed of the humanisation of employed labour? The reality may be more complex. Even if trust itself is indispensable and generally a very positive element in social relationships, in certain circumstances it may become a tool of coercive (self) control, giving rise to the tensions and paradoxes in WFH contexts. To relieve us from, or in fact confirm our intuition, we have conducted empirical research relating responsible autonomy and control to reciprocal trust.

## Methodology

To develop an in-depth understanding of our participants’ complex thoughts, feelings, beliefs and narratives concerning their WFH experiences during the pandemic, and drawing on an interpretivist philosophy (Isaeva et al., 2015), we undertook an abductive qualitative study with a longitudinal aspect among knowledge workers in the UK. Drawing on our participants’ lived experiences allowed us to explore the meanings they attribute to the events happening in the social world around them (Miles et al., 2014). Research participants were selected purposively to provide maximum heterogeneity across different knowledge-intensive industries to access diverse views (Patton, 2015) – see Table 1 for the demographic background information for our sample. Personal networks were used as a starting point to recruit participants and continued through snowballing until theoretical saturation was reached – i.e. the concepts discussed with respondents became highly repetitive and no new aspects were emerging (Saunders and Townsend, 2016). Despite the possible limitation of recruiting similarly minded individuals and excluding some members of the population of interest, a snowballing sampling strategy allowed us to access information-rich, collaborative and insightful informants (Patton, 2015).

**Table 1.** Participant profiles.

Code	Profession	Gender	Age	Interview^a^	Code	Profession	Gender	Age	Interview^a^
P1	Civil servant	F	23	OO	P16	IT consultant	M	37	WO
P2	Civil servant/Director	M	53	OO	P17	Business architect	F	39	WO
P3	Senior adm. mgr.	F	61	OO	P18	Head of research & policy	M	31	WW
P4	Programme manager	F	41	OO	P19	Academic	F	35	WW
P5	Change impl. mgr.	F	47	OO	P20	Banker	F	34	WW
P6	Academic	F	43	OO	P21	Academic	F	40	WW
P7	Academic	F	33	OO	P22	Medical claim manager	F	61	O
P8	Academic	M	55	OO	P23	IT support	M	53	O
P9	Mgmt. consultant	M	42	OO	P24	Researcher	M	30	O
P10	Researcher	F	31	OO	P25	Academic	M	35	O
P11	Mgmt. consultant	F	36	WO	P26	Academic	M	58	O
P12	Mgmt. consultant	M	41	WO	P27	Mgmt. consultant	F	35	W
P13	Business architect	F	43	WO	P28	Mgmt. consultant	F	37	W
P14	Materials coordinator	F	43	WO	P29	Librarian	F	46	W
P15	Project manager	M	58	WO	P30	Social worker	F	38	W

a Interview type: O = oral, W = written. The letter order indicates the type of interview conducted during first and second interviews.

Abbreviations: P = participant; adm. = administration; impl. = implementation; mgmt. = management; mgr. = manager.

Data were collected by the lead author through semi-structured interviews in two separate time periods. The first round of interviews was conducted in May 2020 with 30 participants. Considering the increased family obligations and adaptation to new working conditions at the time, we felt that it was appropriate to give participants the choice of doing oral or written interviews. Fifteen interviews were subsequently conducted via a video-conferencing tool (e.g. Zoom, Messenger) and lasted around an hour. The remaining 15 interviews involved participants responding to the same predetermined set of open-ended questions in written form. We recognise that the written interviews could be restrictive in terms of the inability to follow up on the emerging interesting concepts and discussions. However, they allowed participants to respond to questions at their convenience whenever they had time. In return, we still received long responses to questions and gained rich data, which might not have been possible if we had insisted on oral interviews. Additionally, we contacted our participants completing written interviews for further clarification or elaboration of responses where needed, thereby partially overcoming the limitations of such interviews.

A similar approach was adopted for the second round of interviews a year later in May 2021. Twenty-one interviews were conducted with the same participants, of which 17 were oral and four were written (see Table 1). In the first round of interviews, the overall questions were general, asking participants to reflect on their WFH experiences during the first UK national lockdown and how their employers responded in relation to WFH practices and organisation of working patterns. For example, we asked whether they were able to perform their job fully when WFH, how they would compare their WFH experiences to working at their usual workplace, and what the positive and/or negative learning outcomes were when WFH.

In our second round of interviews in May 2021 (after a further strict lockdown had ended and at which time national restrictions were gradually easing), we asked more focused, specific questions reflecting on the participants’ thoughts on autonomy and possible managerial controls set by employers to monitor remote working. For example, in addition to asking about the changes in the overall workloads, WFH experiences and organisational support of WFH practices, we asked whether they experienced/observed any mechanisms put in place by their employer/managers to control and monitor WFH practices and to what extent they have discretion over their work duties. In line with our abductive approach, these focus areas were determined based on the emergent themes from our first interviews. Having a longitudinal aspect meant that we were also able to compare our participants’ experiences at two different points in time and observe changing feelings, attitudes and perceptions.

**Figure 1.**
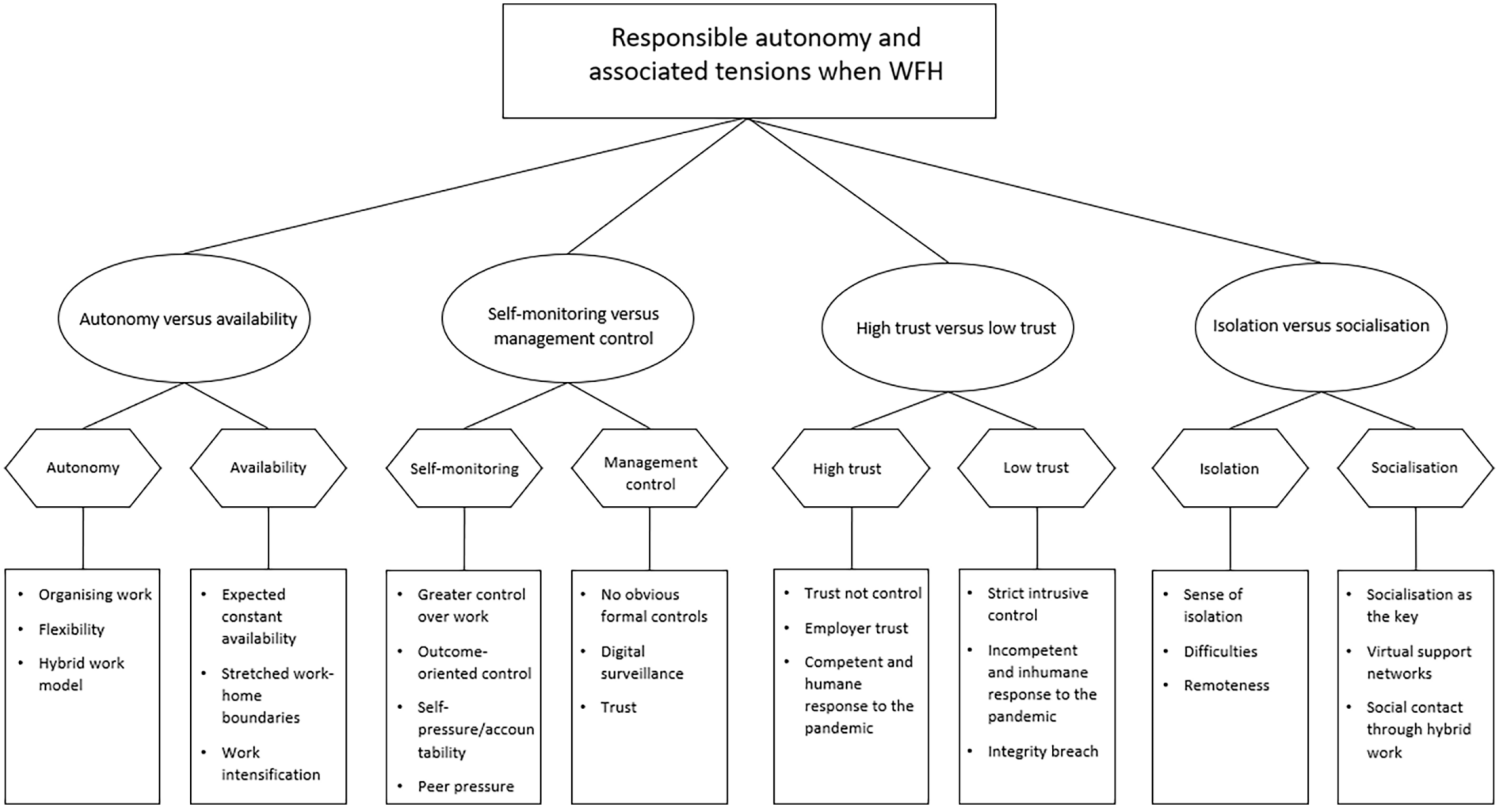
Thematic map.

In order to make sense of the data, we used reflexive thematic analysis (Braun and Clarke, 2022). As part of this method, we identified the semantic, participant-driven codes. We then grouped the codes with similar meanings into themes. Upon reflection on the initial themes, we identified the overarching themes, themes and subthemes (Braun and Clark, 2022). In addition, through an iterative reflexive process, where we considered the relationships across our observations and emergent themes and compared these to the extant theories (Alvesson and Kärreman, 2007), we identified four overarching themes constituting the tensions associated with responsible autonomy and WFH: (1) autonomy versus availability, (2) self-monitoring versus management control, (3) high trust versus low trust and (4) isolation versus socialisation (see Figure 1 for our thematic map). The initial coding was undertaken independently by two of the authors. The development of the final thematic map was reached following all authors deliberating over each theme and discussing the differences until consensus was achieved.

## Findings

The findings are presented below under the four themes relating to tensions. Almost all participants had continued to WFH when we interviewed them a second time in May 2021. Whilst in our initial round of interviews the overall feelings were subjugated by uncertainty, anxiety and fear about the consequences of the pandemic, in the second round of interviews the overall sentiment became more optimistic. Initial broadband/network issues were largely resolved, parenting pressures were eased during periods when schools were open, and our respondents had gained more expertise with digital working and work practices and expectations were clearer, which led participants to report feeling less anxious. Subsequently, work experience was overwhelmingly described as ‘normal/new normal’. Nonetheless, responsible autonomy gained by employees/conceded by employers led our participants to narrate complex stories underlined by contradictions, tensions and paradoxes. We now expound on these tensions.

### Autonomy versus availability

All participants reported having considerable autonomy and discretion over their work and how they work. However, many believed this has increased further during WFH, as they were able to plan their day more independently and flexibly. The issues discussed within this theme were mostly very similar across two rounds of interviews. Participants claimed to be more productive due to organising their own work schedule, achieving more focused work without interruptions, and utilising the time which otherwise would have been spent commuting. Greater autonomy also meant having more flexibility during the day and being able to incorporate non-work activities, including going on walks or exercising, waking up later than usual, having meals and balancing housework and family responsibilities. For example:

Now, I can go mountain biking at the morning before work rather than going on a train for two hours to get to work. (P25, Academic, First interview)

I have the flexibility to do the work when I want to work. I feel freer to do other things for the family, going to the bank, shopping, walking the dogs. . . . You wouldn’t mind having a few hours off in the afternoon and for urgent things you come back to them in the early evening, compensating for the time used. (P15, Project Manager, Second interview)

Although some participants talked of how increased autonomy and discretion contributed to ‘higher quality of life’ and ‘greater work–life balance’, most participants raised concerns about deteriorating work–life balance, using emotive language as they observed that clear boundaries between work and personal life no longer existed:

You live where you work, you work where you live; there is no boundary between the two! (P1, Civil Servant, Second interview)

The challenge of sticking to ‘standard’ working hours was exacerbated by a sense of changed expectations of availability, illustrated by emails arriving outside of standard working hours. Participants said that this reinforced the idea that they should always be contactable:

In the past there was the expectation if you were working from 9 to 5 and anytime within this you would be contactable. Now people think that you are contactable any time of the day or night. (P2, Civil servant/Director, Second interview)

We are expected to be available during normally out [of] office hour[s] and it is difficult to resist since working from home creates a little bit of guilt feeling and we try overcompensating this by usually working more than what we would normally do. I have a great increase in the number of meetings since the start of the lockdown. (P27, Management consultant, First interview)

Especially during the second round of interviews, participants overwhelmingly talked about issues associated with work intensification. In particular, the accounts portrayed situations characterised by increased workloads as individuals spent time adapting their previous working practices (from pre-COVID) to maintain similarly high levels of performance while working from home. Many also talked not just of the effort required to continue to deliver at previous standards, but also of the need to meet changing, and often increasing work demands. Interestingly, some participants opined that remote working was still seen as a benefit or privilege, as often portrayed prior to the pandemic when employee choice was a key factor in WFH; clearly choice was no longer relevant, given governmental mandates for employees to work from home ‘wherever possible’.

There is an outright consensus among my colleagues that the overall workload has increased tremendously. The majority of people are complaining about the lack of respect to personal time, and we are under pressure to be available for extended hours. We are not expected to complain about the job intensification since the ability to work remotely is considered as something like a favour to us granted by our employer. I had to attend meetings way beyond my office hours, and I feel like the concept of working hours has lost its meaning. (P9, Management consultant, Second interview)

In some cases, participants explained that their employers were aware of excessive working hours and the possible adverse impact this may have on staff wellbeing, in addition to other possible stressors which individuals may be facing. This led to advice to take some time for recreational activities. However, for many or our interviewees, this was not feasible due to job obligations and deadlines:

Everyone is working 12 hours in a day. Management asked us to block private time in our calendars but in reality, it was not possible as everyone works in specific projects with very strict deadlines. I am working on Australian time now and start working at 4 a.m. (P13, Business architect, Second interview)

Paradoxically, however, and without exception, all those complaining about workload intensification, stretched work–life boundaries and longer working hours reported wishing to continue WFH or hybrid working. A participant explains this dilemma:

There’s an irony in all of this . . . ironical trade-off; I like working from home . . . it’s a trade-off. There’s more expectation of always being available but more time to do the tasks in a way that I have some control over it. (P8, Academic, Second interview)

### Self-monitoring versus management control

Control-associated issues were predominantly discussed in the second round of interviews, which drives our discussion here. Elements of greater autonomy and discretion over work were associated with experiencing fewer direct control mechanisms set by employers to monitor employees. Consequently, this meant that employees had greater control over their work:

I have a fair degree of autonomy. There is direction set from the partner I work for but in terms of how I get the outcomes they want I have a fair amount of control over them. (P12, Management consultant, Second interview)

Many participants noted not observing any formal control mechanisms. Rather, they talked of delivering work on time as a clear indicator of their productivity, with control being seen as results and output focused. For example, a participant mentioned:

My work is almost self-*assessed* in the way that if I am not doing what I have to do, it is pretty easy to assess that I am not working. (P7, Academic, Second interview)

A senior programme manager explained her approach to managing her staff and expectations regarding outputs:

I am very adamant that what we do is outcome based rather than sitting at our desks, kind of thing. If you get your work done, I am not going to really care how you do it. . . . You know what your workload is, what your deadline is. If you cannot meet it, let me know; otherwise, how you spend your day is entirely up to you. This is how I manage my team; the organisation is very flexible, and outcome oriented as well. . . . The pressure we put on ourselves is purely the pressure we put on ourselves. It is not necessarily the pressure the organisation forced upon us. (P4, Programme manager, Second interview)

Evidently, this autonomy was combined with responsibility (responsible autonomy). Linked to discretion over when they completed their work roles, several participants highlighted their conscientiousness in desiring to do a good job. Consequently, self-motivation to work responsibly and diligently undoubtedly served as a self-control and peer-pressure mechanism.

Feelings of accountability and responsibility relating to responsible autonomy, therefore, partially substituted for direct managerial controls and served as a self-monitoring mechanism. Some participants even talked about feeling guilty if they could not respond to emails when occupied by other activities during a workday. Significantly, some participants remarked that application of strict control mechanisms by management would not be well received by employees. One participant said they would reduce employee wellbeing, job satisfaction and commitment:

There is a level of tolerance and understanding associated with working from home and if the firm was to bring in tight monitoring and controls over working habits and things like that then it would be counter to people’s happiness. There is more of a focus on making people able to work and to some extent enjoy that work as much as possible given some of the hardships people are going through, rather than monitoring and control. (P12, Management consultant, Second interview)

Peer behaviour was also seen to influence colleagues’ work patterns and, in most cases, due to the nature of working in teams, individual success was described as contingent upon group success. Individuals were acutely aware of the hours worked by colleagues, for instance, and they described a sense of obligation to ensure their own performance involved similar hours. As well as this form of self-monitoring, several participants pointed out that their experience of working on project teams with clearly identified interdependencies between elements of the deliverables, means that if anyone fails to deliver, this will be easily observable. Notably, this was discussed at both time periods.

Interestingly, some participants observed that managerial control mechanisms were more often based on new forms of digital and technological control, relating this to being monitored for online availability:

The check is really that you have to be available. There is some control, but it is more informal. Nobody comes and checks if I am working in front of my computer but if they don’t get a hold of me then that would probably be a problem. (P6, Academic, Second interview)

Others talked about being aware of their status (available/not available) being visible in software such as Microsoft Teams or Skype, sensing that their presence was being monitored or tracked, even though this was a feature of the software, rather than something implemented by management intentionally. However, it contributed to what the participant above described as ‘this constant state of readiness that you have to maintain to be always available’. Another participant explained:

In the past we had unmonitored time slots for travel, going out to eat and socialising but now we are expected to be present and online all the time. This puts me in a constant stress of trying to appear online, which wasn’t a concern before. Working remotely made the corporate tracking easier and I feel under more pressure compared to pre-pandemic times. . . . I was in full control of my client engagements and travel arrangements. Now, I need to follow the meeting schedule of my team leaders which reduces my feeling of empowerment. (P9, Management consultant, Second interview)

One participant explained how her manager’s close monitoring of WFH created substantial stress, causing her to resign:

I have changed jobs during the pandemic and one of the main reasons for doing so has been the controlling nature of my previous manager. My previous manager’s style was more overbearing than hands-on, and they were obsessively micromanaging everything. Moving from being in the office to working remotely when the lockdown had started led to being asked to complete timesheets to account for every single hour of the day. It was stressful. (P10, Researcher, Second interview)

Overall, we also discovered that trust substituted for control, especially when formal control mechanisms were not or more accurately could not be in place. We elucidate this further in the subsequent section.

### High trust versus low trust

The extent to which management applies technological control to monitor employee attendance and work performance or to facilitate responsible autonomy affects levels of trust between employees and management. Thus, trust and control assume a substitutive relationship, with increased trust leading to less need for intrusive control. Various participants expressed their experiences of managers trusting them rather than feeling controlled. For example:

I always felt that trust and respect that [my manager] knows that I am doing the work. . . . He works alongside me, doesn’t control me, so he naturally is going to see what I am contributing. (P5, Change implementation manager, Second interview)

Responsible autonomy builds on the foundation of trust and therefore management needs to trust or learn to trust their employees, something which gained much greater significance with the abrupt switch to WFH.

Any micromanagement potential is eliminated. Management is forced to trust their employees if they haven’t trusted them before. (P20, Banker, First interview)

A participant explained the significance of her employer’s trust in her and its impact on her work as follows:

I think it is just the flexibility of the work and trust of your manager and employer makes a big difference to your working day because you are relaxed when you are working. Everyone’s work is busy and can be stressful at times and you need to be able to have that relaxed atmosphere given that you are at home 24 hours in a day. . . . So, for me it is the trust of my employer to be able to get on with my job the way I want to do it during my workday. (P22, Medical claim manager, First interview)

Nonetheless, several participants mentioned historical employer resistance to WFH, which had been shown to be groundless based on the successful achievement of organisational objectives under the current unanticipated circumstances. Participants articulated this, emphasising the importance of trust:

What the last year showed is that we do work, and we can do the work if we are not supervised. So, basically, we can be trusted to do our work wherever we are. (P6, Academic, Second interview)

I believe in the beginning managers did not know what to expect from employees and they lost complete control over what employees are doing, lost visibility. However, as time went on, managers saw that working from home is actually working. People do their job as they normally do, or even better. They learnt to trust. (P20, Banker, Second interview)

Issues associated with trust were discussed significantly in both rounds of interviews. However, the first wave of interviews also comprised discussion of organisational trust based on the organisation’s response to the pandemic. Where employers proved to be capable of managing the crisis competently and humanely, this generated increased levels of trust. Several reasons may apply here, such as the employers’ adherence to safety guidelines, taking a proactive approach to dealing with the pandemic, consulting and communicating with employees clearly and regularly (employee voice), enabling WFH and allowing flexible working hours, offering support for issues like mental health and WFH equipment. All these issues were discussed as contributing to enhancing trust.

Nonetheless, there were also situations where trust was lower, and distrust was evident. Various reasons were highlighted, including concerns about employers not protecting employees’ safety and not listening to employees’ voices (poor communications, inadequate consultation). It is noteworthy that where a lack of integrity was mentioned, for example when senior management used the crisis opportunistically to drive through their own agenda or impose financial cuts even when organisations were financially sound, distrust was particularly high. One participant commented:

The response taken by my organisation was rather extreme in the beginning of the pandemic. They applied harsh measures with multiple redundancies and pay cuts to withstand possible revenue reductions. Those projections were proven wrong and in contrast, the revenue has even increased but the extreme measures were never reversed. I feel like the pandemic was used as an excuse to implement significant cost-cutting measures and this has significantly reduced my trust towards my organisation. As a result, there is a significant reduction in my loyalty towards the organisation which made me think that under the surface of all those shiny words and promises [lies] a great deal of greed and profiteering. (P9, Management consultant, Second interview)

### Isolation versus socialisation

An interesting tension emerged for many participants experiencing a sense of isolation, as they now worked full-time from home, and missing opportunities to socialise in the workplace. In fact, almost all participants identified socialising as the most significant missing element of WFH full-time, referring to daily office activities such as having coffee breaks or lunch with colleagues, meeting up after work hours and so on. Interestingly, whilst all these participants were content with doing the actual work at home, even finding this to be more productive, many discussed socialising as the main reason for wanting to go back to the office, albeit occasionally:

What Covid has underlined to everyone is if you want a desk, if you want to sit somewhere quiet, you might stay at home. Really, the point of going to the office is to meet people, talk to people one-on-one or in big groups. (P2, Civil servant/Director, Second interview)

Participants repeatedly expressed desire for a hybrid work model that incorporates both WFH and office work (e.g. three days at home and two days in the office). Socialisation with colleagues was seen as the key differentiator in the desire for going to the office.

Participants talked about attending virtual social events such as coffee meetings, happy hour celebrations, etc. The value the participants attached to such virtual events was quite positive in the first round of interviews. Some participants also discussed building support networks which brought them closer:

We were maintaining contact a lot and it’s not just formal contact. This encouraged us to be more social. This really pushed us not just to see each other as colleagues supporting one another in a professional basis but supporting us generally. . . . So, we are physically isolated, but in a weird way it brought us closer together emotionally. (P24, Researcher, First interview)

In the long term, however, those virtual events did not reproduce the same desired impact achieved by face-to-face socialising. It was apparent that the novelty of such events wore off, resulting in fewer virtual events being organised. Lack of socialising was increasingly seen to exacerbate the feelings of isolation, especially for those living alone.

Various difficulties with WFH were emphasised in both waves of interviews, including its unfavourability to networking and collaboration or lack of trustworthiness cues:

If you are in the office, you have the usual water bottle/kitchen conversation where you meet new people; if you sit in an office, you just talk to the person sitting next to you – which all stimulates new ideas, collaboration. Working at home makes this more difficult because you lose the spontaneous element of it. (P20, Banker, Second interview)

Signals of trustworthiness are communicated throughout face-to-face interactions, acquiring those in a virtual realm is very difficult; you don’t get the same cues, don’t have the same neuro synchronicity going on. Those are the losses. (P26, Academic, First interview)

When social aspects are removed from the workday, it becomes only about work, which, in turn, is not conducive to identifying with and belonging to the work organisation, and this can contribute to a sense of remoteness. This was particularly apparent in the second set of interviews when participants had been working from home for a considerable length of time with little interaction or communication with their employer and increasing feelings of detachment and being undervalued and vulnerable:

Not being physically present at work makes me feel less valuable and replaceable. Being in the office serves many purposes other than fulfilling our deliverables and the feeling of belonging, networking, team building – they are some of the intangible benefits. (P9, Management consultant, Second interview)

Drawing on our participants’ narratives, it appears that organisational change towards working more flexibly and remotely after the pandemic may be irreversible:

The pandemic put cultural change to fast forward. Things were already happening anyway. Things that might have taken 15 years to change have taken one year. (P2, Civil servant/Director, Second interview)

Why chain people to getting into the factory, the 21st century factory, because it has always been like that? (P15, Project manager, Second interview)

## Discussion

Our study theoretically extends Friedman’s (1977) theorisation of responsible autonomy in the contemporary context of the coronavirus pandemic. We revisited the concept of responsible autonomy by analysing the interplay of employee autonomy, management control and trust experienced by knowledge professionals compelled to work remotely during the pandemic. Unlike Friedman (1977), we do not see employers’ implementation of responsible autonomy as a deliberately intended managerial strategy or resulting from pressures to accommodate worker resistance. Instead, responsible autonomy increased suddenly and rapidly for many workers who were able to WFH during state-enforced lockdowns in countries like the UK, which employers had to comply with. The article adds theoretical insights into the tensions and paradoxes of responsible autonomy affecting knowledge workers.

These tensions and paradoxes were illustrated empirically through qualitative research conducted in two phases (May 2020 and May 2021) during the crisis. Whilst in the first round of interviews the tensions that knowledge professionals experienced centred around anxiety caused by the COVID-19 pandemic and getting used to new working practices experienced by those who did not have the opportunity to work from home in the past, in the second round tensions shifted towards issues associated with work intensification, new digital technological controls, etc.

The main contributions revealed by our research are summarised in more detail below. As stated, Friedman (1977) distinguished between two main managerial control strategies: direct control and responsible autonomy. Our empirical findings relating to knowledge professionals WFH demonstrate the incongruence between responsible autonomy and intrusive control (Thompson, 1983). Management control is a complex dynamic with different layers. Our research supports the analytical observation that it is possible for management to loosen direct control at the point of production or service delivery through devolving responsible autonomy to employees over elements of organising work tasks, and still assume general control and authority at a higher strategic organisational level (Edwards, 1986); for instance, if management focuses on performance outputs rather than presenteeism (Van Dyne et al., 2007). It was notable that this loosening of direct control was forced upon employers in our study during the unique circumstances of a pandemic, and not something they may have voluntarily implemented.

Our findings also highlight the vital role trust plays in responsible autonomy – an overlooked issue within Friedman’s (1977) original labour process theorisation of responsible autonomy, but identified by Fox (1974). Trust is the glue in responsible autonomy. More specifically, successful WFH is highly contingent upon reciprocal trust between employers and employees, best supported by institutionalised trust embedded in good employment relationships (Fox, 1974; Siebert et al., 2015; Yunus and Mostafa, 2022). Fox’s (1974) analysis that high discretion/high trust (responsible autonomy) dynamics are most likely in occupations like professional work is relevant to our research. However, unlike the unitarist accounts of common interests that now dominate trust research, Fox (and ourselves) identify conflicts of interest and tensions that can erode trust and even cause a spiral of distrust.

In line with radical sociology of work scholars like Fox (1974) and Friedman (1977), our participants’ narratives about experiences were underlined by various contradictions and tensions arising from responsible autonomy, but in the contemporary context of the COVID-19 pandemic. Research on FWAs prior to the pandemic reported contradictory outcomes, highlighting the complexity and paradoxes of such arrangements (Putnam et al., 2014). Enforced WFH during the pandemic has further contributed to pre-existing paradoxes and tensions.

Our research contributes to knowledge by identifying four main tensions: autonomy versus availability, self-monitoring versus management control, high trust versus low trust, and isolation versus socialisation. On the one hand, our findings show that responsible autonomy resulted in mutual gains: positive employee experiences of greater task discretion, and higher productivity for employers, weakening the argument of WFH-sceptic employers. On the other hand, our findings are consistent with prior research outlining the ‘autonomy paradox’ – i.e. more autonomy and discretion over organising work coinciding with increased effort, longer working hours and blurred work–life boundaries and a ‘dark side’ of work–life balance for employees (Hassard and Morris, 2022; Kelliher and Anderson, 2010; Mazmanian et al., 2013; Perrigino et al., 2018). Evidently, many of our participants found work intensification and extensification to be a real challenge. Difficulty in separating work life from home life, losing control over working hours and endless online meetings/virtual presenteeism were experienced by employees.

Closely linked to work intensification, expectation of constant availability was a prominent tension associated with increased responsible autonomy. Employees in our research clearly welcomed having significant responsible autonomy and discretion over work, which enabled them to organise their own work schedules and work–life balance. The flip side, however, is that the often-demanding nature of professional knowledge work has always required a high level of availability and accessibility (Cañibano, 2019). For many of our participants, the pressure of always being available, caused by either management, personal expectations or peer pressure, has been reported to significantly extensify work. Indeed, with ‘home’ becoming ‘work’, the concept of standard office hours has arguably been obliterated.

Two other tensions we identified were associated with self-monitoring and managerial control, and different degrees of trust pushing in opposite directions (low trust/distrust–high trust). Employee autonomy, accountability and responsibility among our knowledge-professional participants often substituted for direct managerial controls and served as a self-disciplining and peer pressure mechanism. In turn, this self-discipline helped to build reciprocal trust in employment relationships. However, our findings also demonstrate that, in some situations, continued managerial use of formal direct control and technological surveillance mechanisms could be detrimental, and cause distrust (De Vaujany et al., 2021; Lautsch et al., 2009), even leading, in one example, to an employee resignation. Interestingly, some participants observed a managerial mentality that presented WFH as a privilege to be bestowed on worthy employees or high performers as some sort of reward. Thus, people should be grateful that they were ‘allowed’ to work flexibly. Evidently, there are clear limits to reciprocal trust even for knowledge professionals in high discretion roles – it can be easily damaged (creating a spiral of distrust) by intrusive managerial control, including using new online platforms as a tool for technological monitoring and surveillance.

A further tension revealed in our research is that WFH limits face-to-face socialising, potentially causing feelings of isolation. Socialising is an integral part of overall organisational life, and particularly vital for newcomers (Peltokorpi et al., 2022). WFH was not very conducive to building trust with people newly joining the organisation, and virtual socialising events were not as popular as face-to-face activities.

Interestingly, many participants seemingly accepted tensions and paradoxes as a palatable trade-off for the autonomy of being able to work from home. This was particularly so where management was competent at organising work and humane in its treatment of employee welfare, thereby building reciprocal trust in employment relationships.

## Directions for future research and practice

Whilst not fully generalisable, our conceptualisation, findings and lessons learnt from responsible autonomy and WFH could be transferable in order to inform future analysis and understanding of FWAs or WFH practices. Further research is required to analyse responsible autonomy in different contexts (for example, different occupational groups, sectors and countries) and uncover the tensions and paradoxes of flexible working practices like WFH or hybrid working.

Of particular note is that our findings relating to the dynamics of trust point to an important conceptual issue arising from our research. At one conceptual level, trust is both fundamental in constituting the glue binding responsible autonomy, as well as being intertwined with other tensions we identify. At another conceptual level, trust itself constitutes a tension between low trust (distrust) and high trust pushing in opposite directions. There seems to be a significant dialectical tension at play in our study: high trust is required for responsible autonomy, and yet this can prompt individuals to engage in behaviours reflective of the more negative part of these tensions (work extensification, being available online, self-monitoring, peer pressure, scheduled presenteeism). If not managed properly and/or if management also revert to surveillance and monitoring of employees, this can lead to low trust/distrust. Therefore, trust is both fundamental and perceived as inherently fragile, serving as an example of ‘structured antagonism’ (Edwards, 1986). The role of trust in association with responsible autonomy clearly warrants further investigation.

We also offer practical recommendations for management and other stakeholders. The COVID-19 pandemic continues to change work practices. Some organisations are now questioning the old ways of working and deliberating over transitioning to hybrid work models. Our study offers significant insights for management and other actors into the interplay between responsible autonomy, trust, control and associated tensions. Employers carry great responsibility in managing employee workloads and work patterns. In order to maintain and support employee wellbeing and consent, human resources (HR) professionals need to be actively developing policies, in consultation and negotiation with employees and their representatives, to ensure an acceptable level of work–life balance. Employee voice is crucial for mutual gains and avoiding conflict. A key priority is to prevent work intensification and extensification. As exemplified by the experiences of our participants, strategies (e.g. blocking time for personal activities) might not always be feasible due to strict deadlines or unavoidable client demands. Gimmicky corporate wellness and wellbeing policies that do not grant real solutions to the tensions of work could undoubtedly elicit negative feelings among employees, as can overly intrusive managerial controls (notably technological monitoring and surveillance). Therefore, HR professionals, in consultation with employee representatives, could play a vital role in devising future plans regarding issues like flexible working patterns that create mutual gains for employers and employees, helping to avoid conflict and distrust.

## Conclusion

Our study adds to existing knowledge about the concept of responsible autonomy (Friedman, 1977) by analysing the interplay of employee autonomy, management control and trust experienced by knowledge professionals WFH during the coronavirus pandemic. Drawing on two waves of interviews with knowledge professionals, our research advances theoretical insights into the tensions and paradoxes of responsible autonomy. Our findings suggest that responsible autonomy depends on the foundational glue of reciprocal trust between employers/managers and employees, together with employee self-discipline and accountability supplanting direct managerial controls. Nevertheless, responsible autonomy contains paradoxes, contradictions and tensions associated with employee autonomy (autonomy versus availability), control (self-monitoring versus management control), trust (high trust versus low trust) and further tensions arising from WFH (isolation versus socialisation). Interestingly, however, these paradoxes were generally perceived to be a palatable trade-off for the autonomy of being able to work from home, particularly where employers/management were competent at establishing clear work expectations and treated employees compassionately and humanely. However, where this is not the case, distrust can occur.
